# Low Expression of IL-15 and NKT in Tumor Microenvironment Predicts Poor Outcome of MYCN-Non-Amplified Neuroblastoma

**DOI:** 10.3390/jpm11020122

**Published:** 2021-02-13

**Authors:** Yu-Mei Liao, Tsai-Hsien Hung, John K. Tung, John Yu, Ya-Ling Hsu, Jung-Tung Hung, Alice L. Yu

**Affiliations:** 1 Institute of Stem Cell and Translational Cancer Research, Chang Gung Memorial Hospital at Linkou, Taoyuan 333, Taiwan; p920271@gmail.com (Y.-M.L.); hth0204@gmail.com (T.-H.H.); jktung103@gmail.com (J.K.T.); johnyu@gate.sinica.edu.tw (J.Y.); 2 Program in Translational Medicine, Kaohsiung Medical University, Kaohsiung, and Academia Sinica, Taipei 115, Taiwan; 3 Division of Hematology and Oncology, Department of Pediatrics, Kaohsiung Medical University Hospital, Kaohsiung 807, Taiwan; 4 Institute of Cellular and Organismic Biology, Academia Sinica, Taipei 115, Taiwan; 5 Graduate Institute of Medicine, College of Medicine, Kaohsiung Medical University, Kaohsiung 807, Taiwan; yainghsu@kmu.edu.tw; 6 Department of Pediatrics, University of California in San Diego, San Diego, CA 92103, USA; 7 Genomics Research Center, Academia Sinica, Taipei 115, Taiwan

**Keywords:** neuroblastoma, tumor microenvironment, NKTs, IL-15, immunoscore, prognosis

## Abstract

Immune tumor microenvironment (TME) in neuroblastoma (NBL) contributes to tumor behavior and treatment response. T cells and natural killer (NK) cells have been shown to play important roles in the neuroblastoma TME. However, few reports address the clinical relevance of natural killer T cells (NKTs) and interleukin-15 (IL-15), one of the crucial cytokines controlling the activation and expansion of NK/NKT cells, in NBL. In this study, we examined NKT immunoscores and IL-15 expression in both MYCN-amplified and MYCN-non-amplified NBL to correlate with clinical outcomes such as event-free survival (EFS) and overall survival (OS). From Gene Expression Omnibus (GEO) datasets GSE45480 (*n* = 643) and GSE49711 (*n* = 493), we found that NKT immunoscore and IL-15 expression were both significantly lower in MYCN-amplified NBL, and similar results were observed using our clinical NBL samples (*n* = 53). Moreover, NBL patients (GEO dataset GSE49711 and our clinical samples) with both lower NKT immunoscore and IL-15 expression exhibited decreased EFS and OS regardless of MYCN gene amplification status. Multivariate analysis further showed that the combination of low NKT immunoscore and low IL-15 expression level was an independent prognostic factor for poor EFS and OS in our NBL patients. These findings provide the rationale for the development of strategy to incorporate IL-15 and NKT cell therapy into the treatment regimen for neuroblastoma.

## 1. Introduction

Neuroblastoma (NBL) is a neuroectodermal solid tumor occurring in early childhood with 20~50 cases per million individuals [1]. Disease course and treatment outcome for NBL vary significantly depending on clinical features and molecular risk factors. The International Neuroblastoma Risk Group (INRG) provides a consensus pretreatment classification scheme for risk-stratified therapy according to the stage, age, histology, MYCN (v-myc myelocytomatosis viral related oncogene, neuroblastoma derived (avian)) status, and other genetic aberrations of NBL patients [2]. Clinical outcomes for the low-risk and intermediate-risk groups have been excellent when treated with surgery and limited chemotherapy. However, despite the multi-modality treatment with surgery, intensive chemotherapy, stem cell transplantation, and radiotherapy, prognosis for high-risk patients has remained dismal. While the addition of anti-disialoganglioside (GD2)-based immunotherapy has increased event-free and overall survival in patients with high-risk NBL [3], there is still room for improvement.

New advances in immunotherapy have recognized the contribution of immune tumor microenvironment (TME) to NBL development and progression [4,5]. Most aggressive NBLs have amplification of the MYCN oncogene [6], which is associated with poor survival [7,8], and analysis of TARGET transcriptomic data has revealed that fewer natural killer (NK) and T cells are present in the TME of MYCN-amplified NBL [9]. In particular, MYCN expression has been shown to inversely correlate with the expression of ligands for NK-cell-activating receptors [10], suggesting that MYCN amplification may have an adverse impact on the activation of NK cells, which are important players in antibody-based immunotherapy [11]. In addition, T cells in the TME of NBL, as defined by the presence of CD3^+^, CD4^+^, and CD8^+^ subpopulations, are associated with favorable clinical outcomes in MYCN-amplified NBL [12], and another study has similarly reported that increased cytotoxic tumor infiltration lymphocytes (TILs) in MYCN-non-amplified NBL is associated with the presence of activated NK/T cells and improved outcomes [13].

In comparison, few studies have addressed the role of natural killer T cells (NKTs) in the NBL immune microenvironment. While Metelitsa et al. has reported that the presence of NKTs within the primary tumor site is associated with improved prognosis in patients with NBL [14,15,16]; its prognostic values for MYCN-non-amplified NBL versus MYCN-amplified NBL remain to be elucidated. NKTs are CD1d-restricted in that they can be directly cytotoxic against CD1d^+^ cells [17]. Although the majority of human tumors express low levels of CD1d to escape from NKT cytotoxicity, NKTs have been shown to overcome immune evasion by modulation of the TME via inhibition of tumor-associated macrophages (TAMs) [14].

Immunotherapy with dinutuximab, a monoclonal antibody (mAb) against GD2, in combination with granulocyte–macrophage colony-stimulating factor (GM-CSF) [18] and interleukin-2 (IL-2) [19] has been approved for the treatment for high-risk NBL, as GM-CSF and IL-2 are known to augment antibody-dependent cellular cytotoxicity (ADCC) by granulocyte/monocyte and NK/NKTs, respectively. Recent preclinical studies in murine models have demonstrated that the combination of IL-15 and NKTs enhanced anti-tumor activity against NBL [18,19,20]. Although IL-15 is a key player in NK/NKTs development and homeostatic maintenance [21,22], the role of IL-15 in the NBL TME has not been evaluated. In this study, we examined the expression of NKTs and IL-15 in MYCN-amplified and MYCN-non-amplified NBL and explored their prognostic potentials for NBL.

## 2. Materials and Methods

### 2.1. Data Source and Processing

Gene expression profiles and clinical information were obtained from Gene Expression Omnibus (GEO) databases (GEO accession: GSE49711 and GSE45480), and survival data for GSE49711 were acquired from the R2 platform (https://hgserver1.amc.nl/cgi-bin/r2/main.cgi (accessed on 25 January 2021)). Specifically, GSE49711 contains RNA-seq data from 498 NBL patients, while GSE45480 include microarray data from 649 NBL patients. Cell type enrichment scores for NKTs (NKT immunoscores) were calculated from gene expression data using the xCell web tool developed by Aran et al. (http://xcell.ucsf.edu/ (accessed on 25 January 2021)) [23]. Clinical characteristics of the patients involved in GSE49711 and GSE45480 has been previously described [23].

### 2.2. Neuroblastoma (NBL) Samples

Archival samples of cDNA derived from a previous collection of primary tumors from 53 NBL patients were used for the study [24]. These patients were enrolled in studies between 1986 and 1995, and we obtained the samples through the Children’s Oncology Group, the Pediatric Oncology Group, and the Cooperative Human Tissue Network. The study was conducted according to the guidelines of the Declaration of Helsinki, and approved by the Institutional Review Board of Chang Gung Memorial Hospital, Taiwan (protocol code 201701442A3; 11 December 2017). Clinicopathologic information of the participants were summarized in Table 1 and are representative of the NBL population in general. The 5-year event-free survival (EFS) of these patients was 58.8% with a median follow-up time of 3.1 years, while the 5-year overall survival (OS) of these patients was 62.0% with a median follow-up time of 3.47 years.

### 2.3. Real-Time Reverse-Transcription Polymerase Chain Reaction

Total RNA was isolated from NBL specimens using TRIzol (Invitrogen, Carlsbad, CA, USA), and RNA (1 μg) was converted to cDNA using the High-Capacity cDNA Reverse Transcription Kit (Applied Biosystems, Foster, CA, USA) in accordance with the manufacturer’s protocol. The human IL-15 qPCR primer pair was purchased from OriGene (CAT#: HP233499, Rockville, MD, USA), whereas the Vα24 (5′-CTGGAGGGAAAGAACTGC-3′, 5′-TGTCAGGGAAACAGGACC-3′) and the glyceraldehyde-3-phosphate dehydrogenase (GAPDH) (5′-CCACTCCTCCACCTTT-3′, 5′-ACCACCCTGTTGCTGT-3′) primer pairs were designed as described by Grose et al. [25]. Expression levels of Vα24 and IL-15 were determined via quantitative RT-PCR with the SYBR Green Real-Time PCR Master Mix using the Applied Biosystems 7500 Fast Real-Time PCR System, with GAPDH serving as the endogenous control. In accordance with the manufacturer’s protocol, 10 ng of cDNA template were added for each 10 μL qRT-PCR reaction. The fluorescent signals were analyzed by Applied Biosystems 7500 Software v2.0.6.

### 2.4. Statistical Analysis

Relative levels of Vα24 (a member of the subfamily of the T cell receptor which conserved expressed on NKTs) and IL-15 mRNAs were expressed as −ΔCt after subtracting the Ct of the reference gene, GAPDH, from that of the genes of interest. Their prognostic values were evaluated via receiver operating characteristic (ROC) area under the curve (AUC) analysis, and the Youden index (sensitivity + specificity − 1) was calculated to determine optimal cut-off values for high versus low gene expression levels. Survival curves were plotted using the Kaplan–Meier method, with the log-rank test applied for comparison. The Cox proportional hazards regression model was employed to identify independent prognostic factors. Statistical computations were performed with Prism 7.0 (GraphPad Software, La Jolla, CA, USA) and SPSS V22.0 (IBM, Armonk, NY, USA) software.

## 3. Result

### 3.1. NKTs and IL-15 Expression Were Increased in the Tumor Microenvironment of MYCN-Non-Amplified NBL

**Figure 1:**
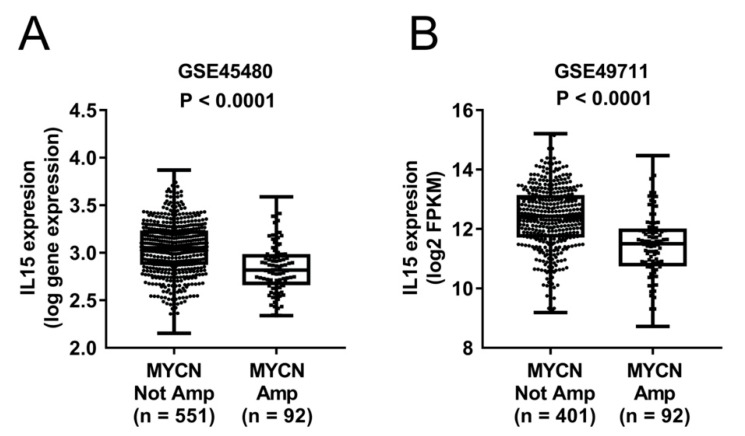
Higher expression of IL-15 in MYCN-non-amplified than MYCN-amplified NBL. IL-15 RNA expression levels were shown as boxplots of log gene expression in GSE45480 (**A**) and log2 fragments per kilobase of exon per million fragments mapped (FPKM) expression in GES49711 (**B**). *t*-test was applied for statistical comparison.

As IL-15-related cell signaling regulates NKT proliferation, differentiation, and survival [26,27,28], we analyzed two large NBL datasets, GSE45480 (*n* = 643) and GSE49711 (*n* = 493), to elucidate the clinical relevance of IL-15 and NKT expression. As shown in Figure 1, IL-15 expression level was significantly higher in MYCN-non-amplified patients than in MYCN-amplified patients in both GSE45480 (median expression value: 3.05 vs. 2.81, *p* < 0.001) (Figure 1A) and GSE49711 (median log FPKM: 12.4 vs. 11.5, *p* < 0.001) (Figure 1B). Consistent with previous reports [16], NKT immunoscores were higher in the MYCN-non-amplified group than the MYCN-amplified group in both GSE45480 (median NKT immunoscore: 0.2494 versus 0.2019, *p* < 0.001) (Supplementary Figure S1A) and GSE49711 (median NKT immunoscore: 0.0761 versus 0.0393, *p* < 0.001) (Supplementary Figure S1B). To validate these findings, we evaluated the expression levels of Vα24 and IL-15 in our 53 NBL clinical samples and found that they were both higher in the MYCN-non-amplified group, although the differences did not reach statistical significance due to small sample size (*p* = 0.15 and 0.19, respectively) (Supplementary Figure S2).

### 3.2. Lower NKT Immunoscore or IL-15 Expression Level Is Associated with Decreased Event-Free and Overall Survival in NBL Dataset

**Figure 2:**
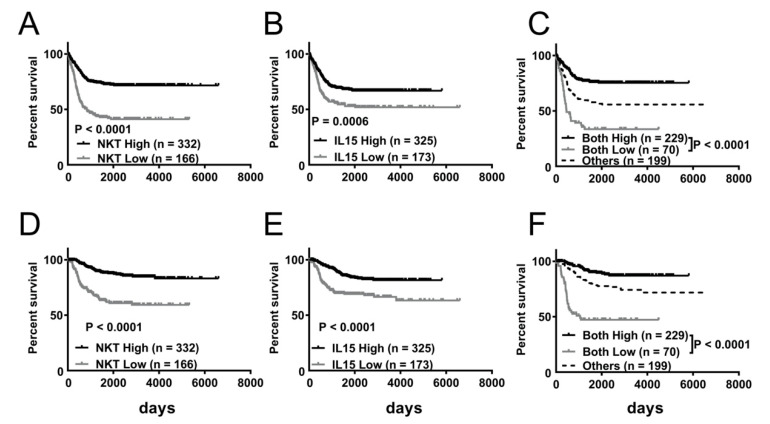
Survival benefit of greater abundance of natural killer T cell (NKT) and IL-15 expression in NBL dataset GSE49711. Kaplan–Meier curves showing event-free survival (EFS) (**A–C**) and overall survival (OS) (**D–F**) of NBL according to NKT immunoscore (**A**,**D**), IL-15 expression (**B**,**E**), and combined NKT immunoscore and IL-15 expression (**C**,**F**). Log-rank test was applied for statistical comparison.

We next examined the prognostic values of the NKT immunoscore and IL-15 expression level using the GSE49711 dataset. Kaplan–Meier survival analysis showed that NBL patients with low NKT immunoscores exhibited significantly poorer EFS (41.2 versus 71.7%; *p* < 0.0001) and OS (63.2 versus 81.6%; *p* < 0.0001) than those with high NKT immunoscores over the course of follow-up (Figure 2A,D). Similarly, a low level of IL-15 expression was significantly associated with decreased EFS (51.9 versus 66.8%, *p* = 0.0006) and OS (63.2 versus 81.6%, *p* < 0.0001) compared to high level of IL-15 expression (Figure 2B,E). To further evaluate the combined effect of the NKT immunoscore and IL-15 expression level, patients were stratified into three groups: both-high, both-low, and others, for Kaplan–Meier survival analysis. As shown in Figure 2C, patients in the both-low cohort had significantly inferior EFS than those in the both-high group (*p* < 0.0001) at 1 year (62.5 vs. 90.2%), 3 years (37.4 vs. 77.9%), and 5 years (33.5 vs. 75.9%). In addition, patients with both low NKT immunoscore and IL-15 expression had significantly poorer OS than those in the both-high group (*p* < 0.0001) at 1 year (79.9 vs. 99.5%), 3 years (47.2 vs. 94.1%), and 5 years (47.2 vs. 89.4%) (Figure 2F).

### 3.3. Survival Benefit of Greater Abundance of NKT Alone or in Combination with Higher IL-15 Expression in MYCN-Non-Amplified NBL

**Figure 3:**
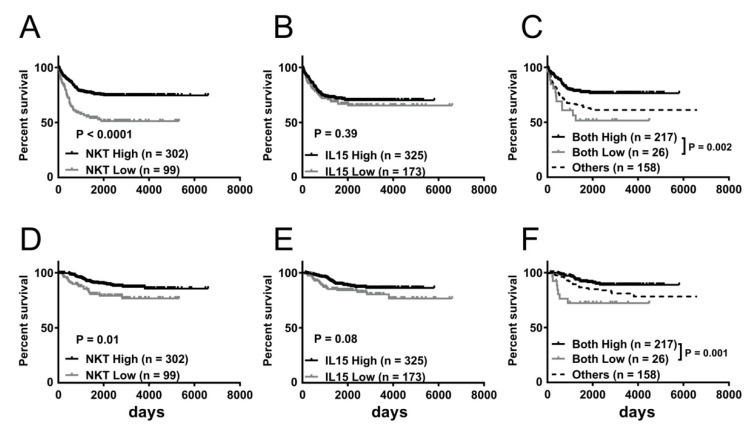
Survival benefit of greater abundance of NKT and IL-15 expression in MYCN-non-amplified NBL dataset GSE49711. Kaplan–Meier curves showing EFS (**A–C**) and OS (**D–F**) of MYCN non-amplified NBL according to NKT immunoscore (**A**,**D**), IL-15 expression (**B**,**E**), and combined NKT immunoscore and IL-15 expression (**C**,**F**). Log-rank test was applied for statistical comparison.

Furthermore, we examined the prognostic values of the NKT immunoscore and IL-15 expression level in MYCN-non-amplified patients from GSE49711. Kaplan–Meier survival analysis showed that MYCN-non-amplified NBL patients with low NKT immunoscores had a significantly inferior EFS (51.2 versus 74.5%; *p* < 0.0001) and OS (76.7 versus 85.6%; *p* = 0.01) compared to those with high NKT immunoscores (Figure 3A,D). Similar trends of association between lower IL-15 expression and worse EFS and OS were noted although not statistically significant (*p* = 0.39 and 0.08, respectively) (Figure 3B,E). To further investigate the combined effect of the NKT immunoscore and IL-15 expression level in the MYCN-non-amplified subgroup, patients were again stratified into three groups as mentioned above for Kaplan–Meier survival analysis. As shown in Figure 3C, both-low patients exhibited significantly shorter EFS than those in the both-high group (*p* = 0.002) at 1 year (76.9 vs. 90.7%), 3 years (60.9 vs. 79.3%), and 5 years (51.5 vs. 77.2%). In addition, these patients with concurrently low NKT immunoscores and IL-15 expression level had significantly lower OS than those in the both-high group (*p* = 0.001) at 1 year (92.3 vs. 100%), 3 years (72.2 vs. 96.1%), and 5 years (72.2 vs. 91.2%) (Figure 3F).

### 3.4. Lower Expression of Vα24 or IL-15 Is Associated with Decreased Overall Survival in NBL Patients

**Figure 4:**
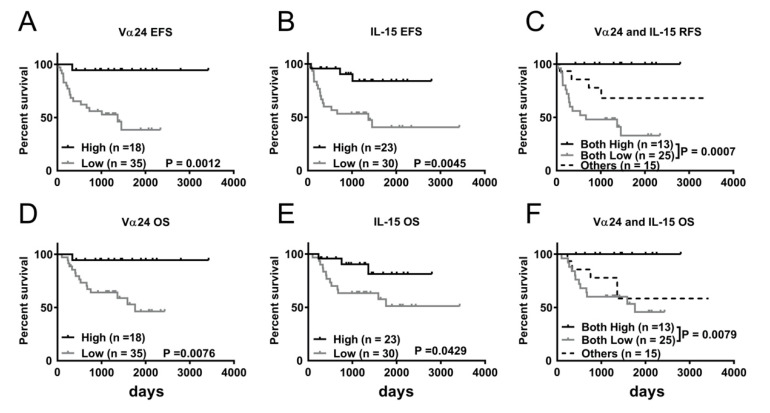
Survival benefit of greater abundance of NKT and IL-15 expression in tumor tissue of NBL patients. Kaplan–Meier curves showing EFS (**A**–**C**) and OS (**D**–**F**) of 53 NBL patients according to Vα24 (**A**,**D**), IL-15 (B,E), and IL-15 + Vα24 combined (**C**,**F**) expression. Log-rank test was applied for statistical comparison.

To validate the results of our in silico analysis of transcriptomic data, we performed correlative analysis between expression levels of Vα24 and IL-15 from 53 primary NBL samples and the patients’ clinical outcome. Kaplan–Meier survival analysis revealed that NBL patients with low Vα24 expression had significantly decreased EFS (38.4 versus 94.4%; *p* = 0.0012) and OS (46.3 versus 94.4%; *p* = 0.0076) compared to those with high Vα24 expression (Figure 4A,D). Similarly, low IL-15 expression was significantly associated with inferior EFS (40.6 versus 83.9%; *p* = 0.0045) and OS (51.1 versus 81.3%, *p* = 0.042) compared to high IL-15 expression (Figure 4B,E). As in our in silico analysis, the combined prognostic impact of Vα24 and IL-15 expression levels was determined by stratifying patients for Kaplan–Meier survival analysis. Our results showed that patients with concurrently low Vα24 and IL-15 expressions had significantly lower EFS (32.9 versus 100%; *p* = 0.0007) and OS (45.7 versus 100%; *p* = 0.0079) than those in the both-high group (Figure 4C,F).

Furthermore, univariate Cox regression analysis of various parameters associated with EFS and OS in NBL revealed that in addition to well-known prognostic factors such as INSS stage, risk category, and age at diagnosis, low expression of Vα24 (*p* = 0.012), low expression of IL-15 (*p* = 0.011), and concurrently low expressions of Vα24 and IL-15 (*p* = 0.003) were all found to be significant predictors of poor EFS (Table 2). In addition, low expression of Vα24 (*p* = 0.03) and concurrently low expressions of Vα24 and IL-15 (*p* = 0.03) were also significant predictive factors of poor OS (Table 3). However, upon multivariate Cox regression analysis, the patient’s risk category (*p* = 0.003 and 0.015, respectively) and low expression of IL-15 (*p* = 0.012 and 0.027, respectively) in combination with low expression of Vα24 remained the only independent predictors of EFS and OS (*p* = 0.009 and 0.045, respectively) (Table 2 and Table 3). 

### 3.5. Lower Expression of Vα24 or IL-15 Is Associated with Inferior Overall Survival in MYCN-Non-Amplified NBL Patients

**Figure 5:**
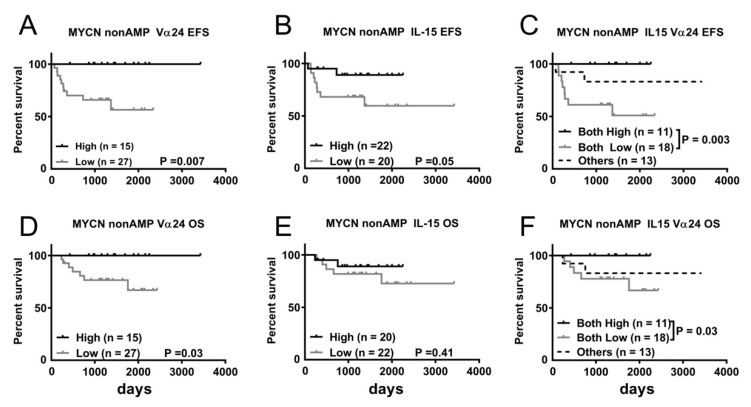
Survival benefit of greater abundance of Vα24 and IL-15 expression in tumor tissues of MYCN-non-amplified NBL patients. Kaplan–Meier curves showing EFS (**A**–**C**) and OS (**D**–**F**) of 53 MYCN-non-amplified NBL patients according to IL-15 (**A**,**D**), Vα24 (**B**,**E**), and IL-15 + Vα24 combined (**C**,**F**) expression. Log-rank test was applied for statistical comparison.

We next evaluated the prognostic effects of Vα24 and IL-15 expression levels in MYCN-non-amplified NBL patients. Kaplan–Meier survival analysis indicated that MYCN-non-amplified NBL patients with low Vα24 expression had significantly worse EFS (56.4 versus 100%; *p* = 0.007) and OS (66.9 versus 100%; *p* = 0.03) than those with high Vα24 expression (Figure 5A,D). Low IL-15 expression was associated with poorer EFS with borderline significance but was not significantly associated with OS (*p* = 0.05 and 0.41, respectively) compared to high IL-15 expression (Figure 5B,E). We also evaluated the combined prognostic value of Vα24 and IL-15 expression levels by stratifying MYCN-non-amplified NBL patients into three groups as described above for Kaplan–Meier survival analysis. As shown in Figure 5C,F, patients with concurrently low Vα24 and IL-15 expressions tend to have inferior EFS (50.9 versus 100%; *p* = 0.003) and OS (66.6 versus 100%; *p* = 0.003) compared to those in the both-high group.

### 3.6. Lower NKT Immunoscore Is Associated with Inferior Overall Survival in MYCN-Amplified NBL Dataset

Kaplan–Meier survival analysis of MYCN-amplified NBL patients in GSE49711 showed that those with low NKT immunoscores had significantly worse OS than their high NKT immunoscore counterparts (30.0 versus 57.0%; *p* = 0.03), although there was only a trend for shorter EFS (*p* = 0.23) (Supplementary Figure S3D). The low IL-15 expression group showed a similar trend for adverse EFS and OS (*p* = 0.15 and 0.20, respectively) (Supplementary Figure S3B,E). Patients who were low for both NKT immunoscores and IL-15 expression level had significantly worse OS than those in the both-high group (*p* = 0.03) at 1 year (70.9 vs. 84.5%), 3 years (26.9 vs. 73.5%), and 5 years (26.9 vs. 71.3%) (Supplementary Figure S3F). As shown in Supplementary Figure S3C, there was a similar trend for the worse EFS in the both-low group (*p* = 0.13).

## 4. Discussion

In this study, we demonstrated that lower expression of both NKTs (Vα24) and IL-15 is significantly associated with poor EFS and OS in NBL patients as well as those in the MYCN-non-amplified NBL subgroup. Moreover, in MYCN-amplified NBL, lower expression of both NKTs and IL-15 significantly correlates with worse OS. These findings provide novel insights for the risk stratification of NBL and suggest that strategies to enhance either NKTs or IL-15 may improve clinical outcomes for high-risk NBL. In addition, association of MYCN amplification with advanced tumor stage and disease progression in NBL has long been known [7,29]. Studies of the MYCN transgenic mouse model have shown that MYCN is sufficient to drive murine NBL tumorigenesis [30]. MYCN is a multifaceted transcription regulator in NBL that can activate genes sustaining growth and repress genes driving differentiation [31]. Furthermore, MYCN in NBL could influence immune surveillance by modulating the infiltration of T cells [9,32], NK cells [10], and NKTs [15], thus supporting the role of MYCN as an immunosuppressive oncogene in high-risk NBL patients. Although MYCN is distinct from MYC (c-myc) [33,34], they are known to share prominent but incomplete redundancy [31]. Recent studies indicate that MYC not only acts as an oncogene but also directly regulates immune responses by various mechanisms that facilitate immune evasion and immunosuppression [35,36,37]. However, whether MYCN in NBL exerts a similar effect on immune regulation as MYC does in other cancers remains to be investigated.

There is currently no report on the clinical relevance of IL-15 in NBL. We are the first to show that lower IL-15 expression is significantly associated with worse EFS and OS in NBL patients. This is in line with previous reports that IL-15 is an independent prognostic marker for patients with prostate cancer [38], breast cancer [39], and lung adenocarcinoma [40]. We are also the first to report the association between IL-15 and MYCN, although the molecular mechanism remains obscure. IL-15 and IL-2 are known to have similar biologic properties due to shared receptor signaling components (IL-2 receptor β and γc), but they differ in their distinctive high-binding-affinity α-chain receptors [41]. While the combination of IL-2 and anti-GD2 has been shown to enhance NK cell-mediated ADCC against NBL [42], IL-2-related toxicities remain a clinical challenge [3]. Compared to IL-2, IL-15 does not provoke activation-induced cell death (AICD), induces less regulatory T cell expansion, and is not associated with capillary leak syndrome [43]. Given these advantages, IL-15 has been used as a monotherapy or add-on strategy for cancer treatment [44,45]. The substitution of IL-15 for IL-2 has led to significant preclinical antitumor activity that exceeded the effect of IL-2 in an orthotopic patient-derived xenograft (PDX) model of NBL [20], and co-expression of IL-15 with GD2-specific chimeric antigen receptor (CAR) has enhanced the therapeutic efficacy of CAR-NKTs or CAR-Ts in xenograft mouse models of NBL [18,46]. Taken together, the demonstrated benefit of combining IL-15 with immunotherapy is in line with our findings that high IL-15 expression significantly correlates with better clinical outcomes in NBL patients.

In a study of 98 NBL patients, NKT level as assessed by Vα24 expression is higher in MYCN-non-amplified than MYCN-amplified cases, with higher expression associated with better clinical outcomes [16]. However, the prognostic significance of IL-15 expression in NBL has not been explored. Low levels of NKTs in the TME are associated with worse outcome in this study, which is in agreement with previous reports [16,47] and in line with the cold tumor hypothesis [5] that lack of infiltrating NKTs in TME predicts poor outcome in NBL. However, this is the first study to further analyze NKT expression in the NBL TME according to the tumor’s MYCN status, demonstrating that fewer NKTs were associated with worse EFS and OS in MYCN-non-amplified NBL and with worse OS in MYCN-amplified NBL. Although little is known regarding the interaction between NKTs and MYCN in NBL, Metelitsa et al. has shown that MYCN regulates the localization of NKTs to the site of disease in NBL and that NKT infiltration is preferentially found in MYCN-non-amplified NBL in a CCL2-dependent manner [16]. NKTs have the capacity to mount strong anti-tumor responses and have thus become a major focus in the development of effective cancer immunotherapy. It has been reported that IL-15 protects antigen-activated NKTs from suppression by tumor-conditioned hypoxic tumor-associated macrophages [48]. Moreover, IL-15 can expand and activate NK, NKTs, and memory CD8^+^ T cells in TME, leading to tumor destruction [49]. The positive impacts of IL-15 on NK and T cells, in addition to NKT cells, may account for the greater power of combined NKT and IL-15 in predicting outcome of MYCN-non-amplified NBL. Novel strategies to enhance NKT activity by the incorporation of IL-15 in GD2-based immunotherapy, such as anti-GD2 antibody or GD2-specific CAR-Ts or CAR-NKs, are being actively pursued in a phase 1 trial of autologous NKTs engineered to co-express a GD2-specific CAR with IL-15 in three children with relapsed or resistant NBL (NCT03294954). Currently, no dose-limiting toxicities have been observed, and objective response with regression of metastatic bone lesions has been noted in one patient [50]. However, in view of the extremely high cost of CAR-Ts, CAR-NKs, and CAR-NKTs cell therapies, the combination of NKT activators with IL-15 might offer a less expensive alternate approach. We previously reported α-galactosyl ceramide (α-GC) and its analogs as strong activators of the anti-cancer effects of NKTs [51,52], and combining α-GC or its analog with IL-15 may be a promising add-on approach for anti-GD2 immunotherapy in high-risk NBL.

## 5. Conclusions

This study shows that in NBL patients with MYCN-non-amplified tumors, lower expression of NKTs (Vα24) or IL-15 alone or in combination are independent prognostic factors for poor EFS, and lower expression of IL-15 alone or in combination with low NKTs (Vα24) are independent predictors for worse OS. These findings support the incorporation of IL-15 into various immunotherapeutic strategies for the treatment of neuroblastoma.

## Figures and Tables

**Table 1:** Clinical and pathological characteristics of 53 neuroblastoma (NBL) patients.

Characteristics	Number (%)
Age at diagnosis	
<1.5 years	32 (60.4)
≥1.5 years	21 (39.6)
INSS stage	
1	7 (13.2)
2	10 (18.9)
3	10 (18.9)
4	20 (37.7)
4s	6 (11.3)
Risk group	
Low and intermediate	30 (56.6)
High	23 (43.4)
Histology ^a^	
Favorable	19 (35.8)
Unfavorable	13 (24.5)
MYCN	
Non-amplified	42 (79.2)
Amplified	11 (20.7)
Progression/Event	
No	34 (64.2)
Yes	19 (35.8)
Death from disease	
Alive	37 (69.8)
Dead	16 (30.2)

^a^ Data not available in 21 patients.

**Table 2:** Cox regression analyses of the various factors associated with event-free survival in NBL patients (*n* = 53).

Overall Survival			
Variables	HR (95% CI)	Favorable/Unfavorable	*p*
Univariate analysis			
Stage	7.64 (2.42–24.15)	1, 2, 3, 4S/4	**0.001**
Risk group	31.15 (4.09–237.38)	Low, Middle/High	**0.001**
Age at diagnosis_1.5	5.08 (1.63–15.79)	<1.5 years/≥1.5 years	**0.005**
Vα24	9.51 (1.26–72.03)	Low/High	**0.03**
IL-15	3.39 (0.97–11.93)	Low/High	0.05
Vα24 and IL-15	3.55 (1.14–11.01)	All Low/Others	**0.03**
Multivariate analysis			
Stage	5.04 (0.49–51.34)	1, 2, 3, 4S/4	0.17
Risk group	95.60 (4.59–189.12)	Low, Middle/High	**0.003**
Age at diagnosis_1.5	1.62 (0.47–5.59)	<1.5 years/≥1.5 years	0.45
Vα24	6.21 (0.80–48.09)	Low/High	0.08
IL-15	4.18 (1.18–14.80)	Low/High	**0.027**
Vα24 and IL-15	8.14 (1.05–63.06)	All Low/Others	**0.045**

Abbreviations: HR, hazard ratio; 95% CI, 95% confidence interval.

**Table 3:** Cox regression analyses of the various factors associated with overall survival in NBL patients (*n* = 53).

Event-Free Survival			
Variables	HR (95% CI)	Favorable/Unfavorable	*p*
Univariate analysis			
Stage	3.69 (1.43–9.49)	1, 2, 3, 4S/4	**0.007**
Risk group	6.63 (2.18–20.15)	Low, Middle/High	**0.001**
Age at diagnosis_1.5	3.85 (1.46–10.17)	<1.5 years/≥1.5 years	**0.006**
Vα24	13.24 (1.76–99.68)	Low/High	**0.012**
IL-15	5.00 (1.46–17.21)	Low/High	**0.011**
Vα24 and IL-15	5.50 (1.81–16.6)	All Low/Others	**0.003**
Multivariate analysis			
Stage	8.51 (0.83–87.84)	1, 2, 3, 4S/4	0.07
Risk group	21.14 (1.79–249.09)	Low, Middle/High	**0.015**
Age at diagnosis_1.5	2.43 (0.73–8.08)	<1.5 years/≥1.5 years	0.147
Vα24	10.26 (1.33–79.36)	Low/High	**0.026**
IL-15	4.95 (1.43–17.12)	Low/High	**0.012**
Vα24 and IL-15	15.55 (1.99–121.41)	All Low/Others	**0.009**

Abbreviations: HR, hazard ratio; 95% CI, 95% confidence interval.

## Data Availability

The GEO data presented in this study are openly available in GSE49711 and GSE45480. The archival sample data presented in this study are available on request from the corresponding author. The data are not publicly available due to ethical.

## Supplementary Materials

The following are available online at https://www.mdpi.com/2075-4426/11/2/122/s1, Table S1: Relationships between the expression levels of Vα24 and IL-15 and various clinicopathologic characteristics, Figure S1: The relative abundance of NKT subtype was greater in MYCN-non-amplified NBL, Figure S2: Expression levels of Vα24 and IL-15 in NBL tissues, Figure S3: Kaplan–Meier curves showing EFS and OS of MYCN-amplified NBL according to NKT immunoscore and IL-15 expression in GSE49711.
