# Supplementary material for: Low Expression of IL-15 and NKT in Tumor Microenvironment Predicts Poor Outcome of MYCN-Non-Amplified Neuroblastoma
Source: J Pers Med. 2021 Feb 13;11(2):122. doi: 10.3390/jpm11020122 (PMC7918138; doi:10.3390/jpm11020122)
Supplement: Supplementary file 1 [file jpm-11-00122-s001.zip › supple Table S1.docx]

**Supplementary Table S1.** Relationships between the expression levels of Vα24 and IL-15 and various clinicopathologic characteristics

| characteristics | Cases | Vα24 | | |  | IL-15 | | |
| --- | --- | --- | --- | --- | --- | --- | --- | --- |
|  |  | Low | High | P |  | Low | High | P |
| Age at diagnosis |  |  |  |  |  |  |  |  |
| <1.5 years | 32 (60.4) | 22 | 10 | 0.76 |  | 18 | 14 | 1 |
| ≧1.5 years | 21 (39.6) | 13 | 8 |  |  | 12 | 9 |  |
| INSS stage |  |  |  |  |  |  |  |  |
| 1 | 7 (13.2) | 4 | 3 | 0.67 |  | 5 | 2 | 0.68 |
| 2 | 10 (18.9) | 7 | 3 |  |  | 8 | 2 |  |
| 3 | 10 (18.9) | 4 | 6 |  |  | 3 | 7 |  |
| 4 | 20 (37.7) | 17 | 3 |  |  | 13 | 7 |  |
| 4s | 6 (11.3) | 3 | 3 |  |  | 1 | 5 |  |
| Risk group |  |  |  |  |  |  |  |  |
| Low and intermediate | 30 (56.6) | 17 | 13 | 0.15 |  | 16 | 14 | 0.78 |
| High | 23 (43.4) | 18 | 5 |  |  | 14 | 9 |  |
| Histology^a^ |  |  |  |  |  |  |  |  |
| Favorable | 19 (35.8) | 14 | 5 | 0.36 |  | 10 | 9 | 0.73 |
| Unfavorable | 13 (24.5) | 12 | 1 |  |  | 8 | 5 |  |
| MYCN |  |  |  |  |  |  |  |  |
| Non-amplified | 42 (79.2) | 27 | 15 | 0.73 |  | 22 | 20 | 0.31 |
| Amplified | 11 (20.7) | 8 | 3 |  |  | 8 | 3 |  |
| Progression/Event |  |  |  |  |  |  |  |  |
| No | 34 (64.2) | 17 | 17 | 0.001 |  | 14 | 20 | 0.004 |
| Yes | 19 (35.8) | 18 | 1 |  |  | 16 | 3 |  |
| Death from disease |  |  |  |  |  |  |  |  |
| Alive | 37 (69.8) | 20 | 17 | 0.005 |  | 17 | 20 | 0.03 |
| Dead | 16 (30.2) | 15 | 1 |  |  | 13 | 3 |  |

^a^Data not available in 21 patients
