# Supplementary figures and images for: Low Expression of IL-15 and NKT in Tumor Microenvironment Predicts Poor Outcome of MYCN-Non-Amplified Neuroblastoma
Source: J Pers Med. 2021 Feb 13;11(2):122. doi: 10.3390/jpm11020122 (PMC7918138; doi:10.3390/jpm11020122)

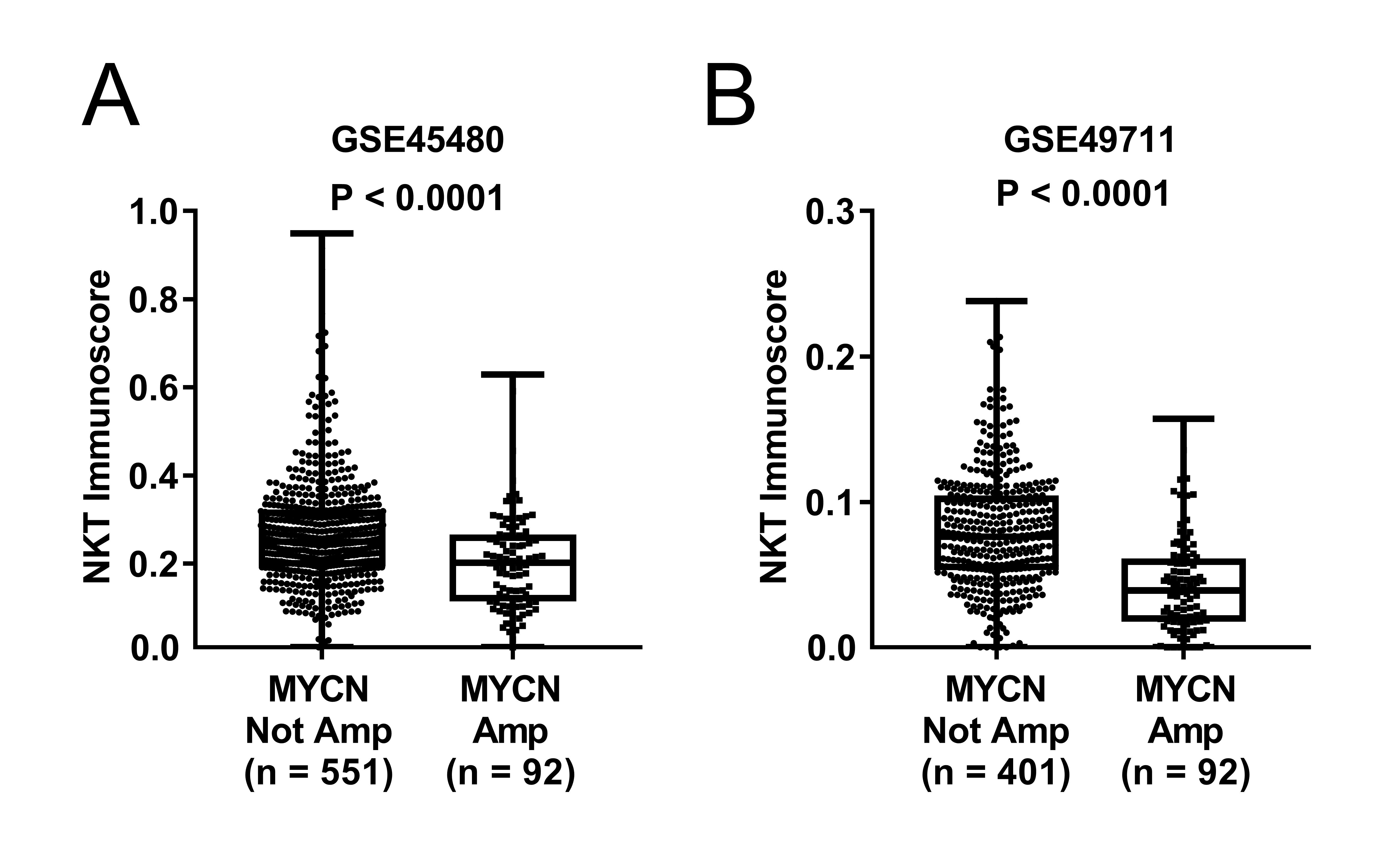

Supplement: Supplementary file 1 [file jpm-11-00122-s001.zip › supple figure1.jpg]

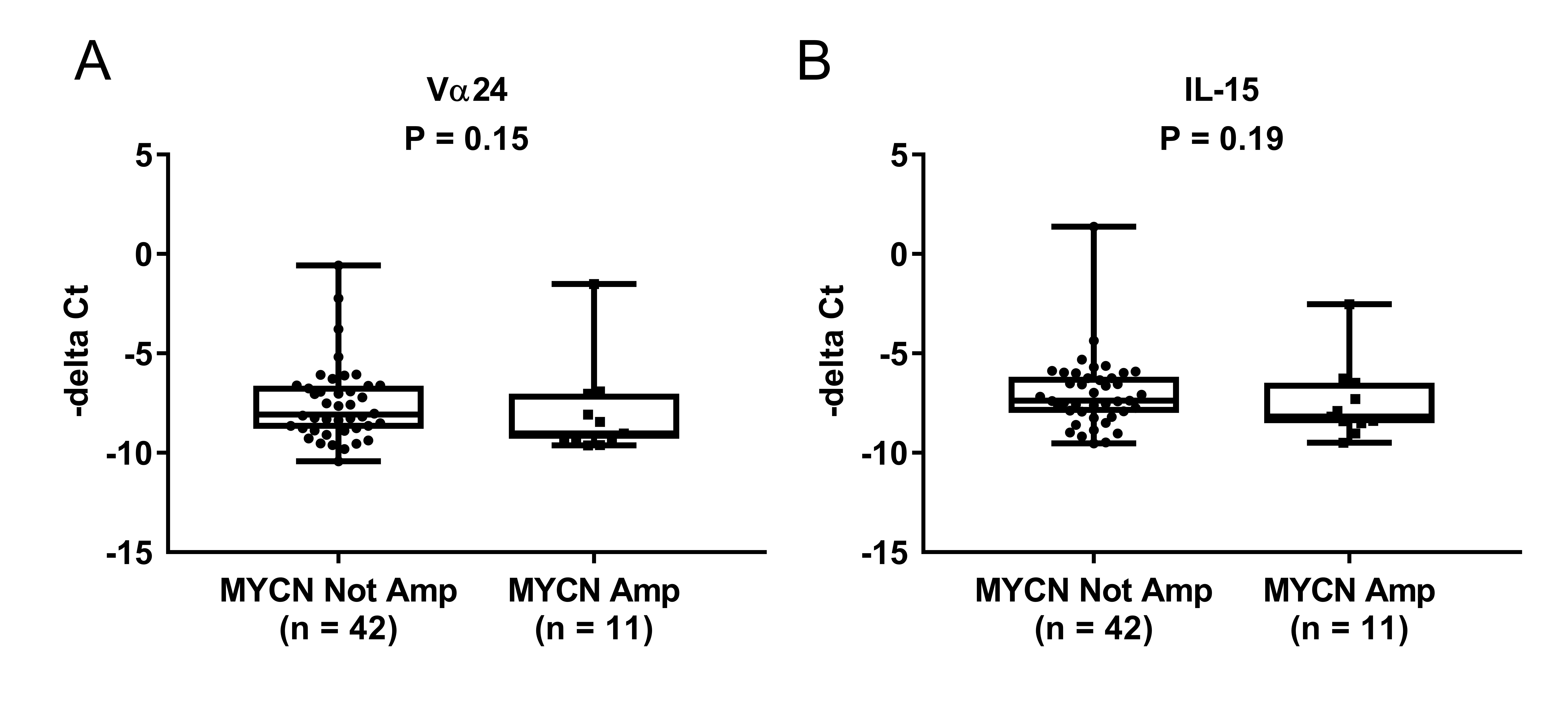

Supplement: Supplementary file 1 [file jpm-11-00122-s001.zip › supple figure2.jpg]

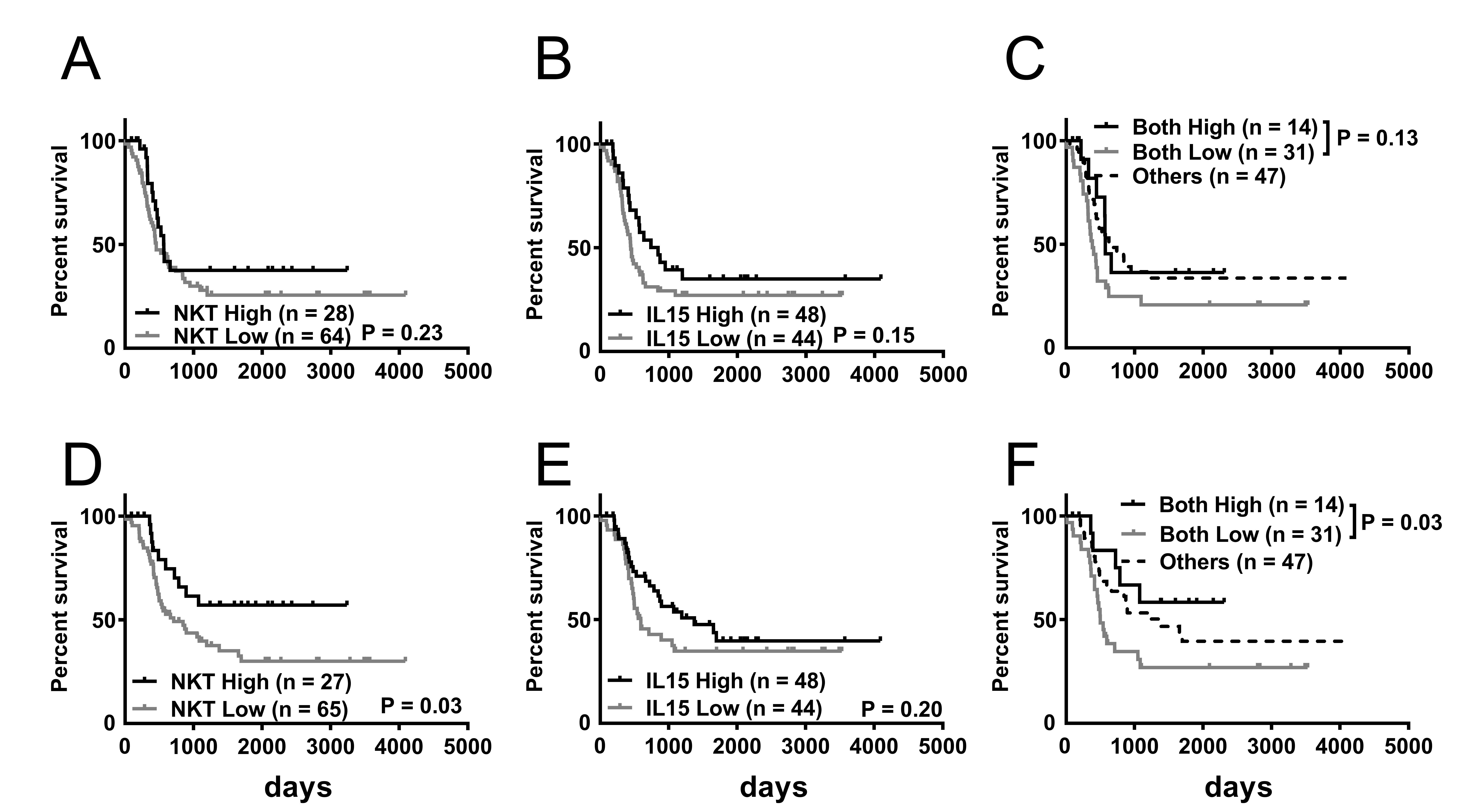

Supplement: Supplementary file 1 [file jpm-11-00122-s001.zip › supple figure3.jpg]
